# Association between housing and health of refugees and asylum seekers in Germany: explorative cluster and mixed model analysis

**DOI:** 10.1186/s12889-021-12458-1

**Published:** 2022-01-08

**Authors:** Verena Dudek, Oliver Razum, Odile Sauzet

**Affiliations:** 1 Department of Epidemiology and International Public Health, School of Public Health, Bielefeld University, P. O. Box 100131, 33501 Bielefeld, Germany; 2 Chair of Data Analysis, Department of Business Administration and Economics, Bielefeld University, Bielefeld, Germany

**Keywords:** Housing, accommodation, refugees, asylum seekers, mental health, physical health

## Abstract

**Background:**

Accommodation for asylum seekers and refugees (ASR) in Germany differs in many ways depending on a range of political, structural, social, and environmental factors. These contextual differences present a challenge for assessing health impacts of refugee accommodation. We aimed to devise a broad typology of refugee accommodation that allows to assess associations between housing and health of ASR.

**Methods:**

We performed a cluster analysis of population-based, cross-sectional secondary data in Germany to identify clusters of refugee accommodation. We then assessed health disparities across clusters by performing bivariate analysis and linear mixed model regression analysis.

**Results:**

We identified four clusters, three of them reflected different types of private accommodation and one pointed to collective accommodation. The collective accommodation cluster clearly differed from the private accommodation clusters in terms of space, area, level of restrictions, social connections and respondent satisfaction. Across private accommodation clusters we also found differences in space, area, and level of restrictions. In regression analysis, belonging to one of the private accommodation cluster was significantly associated with better mental health compared to belonging to the collective accommodation cluster. Physical health was significantly lower in one private accommodation cluster characterized by poor access to public transport and a higher level of restrictions compared to a private accommodation cluster showing better connections and a lower level of restrictions.

**Conclusion:**

We demonstrate that unfavourable conditions cluster in collective accommodation with negative outcomes for mental health but not for physical health. We also found health disparities across types of private accommodation. We conclude that housing plays a role in the production of health inequalities in ASR but needs to be assessed in a differentiated, multidimensional way.

**Supplementary Information:**

The online version contains supplementary material available at 10.1186/s12889-021-12458-1.

## Introduction

The way asylum seekers and refugees (ASR) are housed, i.e., their accommodation, is a postmigration risk factor impacting health: a higher satisfaction with housing conditions has been shown to be associated with lower risks of depressive symptoms among ASR [1]. Poor postmigration socioeconomic and sociocultural living conditions are associated with psychopathology [2] and living in collective accommodation settings is associated with increased risk of mental distress and lower individual’s life satisfaction [3, 4]. However, empirical research has often focussed on only selected, single components of housing through which the overall housing context of ASR is not mirrored adequately.

The housing situation of ASR in resettlement countries is subject to substantial variabilities in terms of political factors determining the housing situation, structural factors of the dwelling, and other factors of the physical and social environment. Accommodation for ASR is predetermined by legal frameworks at different spatial levels (e.g., federal states, districts municipalities). In Germany, for example, residence in state-mandated reception centres is compulsory until refugee status is granted, or 18 months have passed (see §47 Asylum Act). But even for recognized refugees or beneficiaries of subsidiary protection certain residence restrictions may apply [5]. Living in private accommodation is generally possible but access is limited, depending on the asylum status, the willingness and ability of municipalities to reallocate ASR to private accommodation as well as on the local housing market [6].

Structural conditions such as building type, dwelling size or layout also determine the living conditions of ASR. In some facilities, residents share kitchens and bathrooms with all other residents. Other facilities, in contrast, offer separate living units [7]. Some temporary facilities have been set up using residential containers while for other facilities former schools, offices or hotels have been rededicated [8]. Depending on availability and affordability, private accommodation settings also differ, ranging from apartment blocks of varying size to detached houses. Many ASR in private accommodation settings perceive their living space as too small [6].

Area characteristics matter as well for housing satisfaction. Many ASR would prefer to live in urban areas if they could decide for themselves [6], presumably because of the accessibility of important amenities or public transport. If ASR are accommodated remotely and distant from infrastructure, they feel isolated and find it difficult to connect with members of the local population. Approximately 20% of ASR in collective facilities lived in non-residential, industrial areas in 2016 [8] which may have negative consequences for their social integration.

The relationship between refugee accommodation and health cannot be limited to merely distinguishing between private and collective accommodation. A typology of refugee accommodation also needs to consider housing-related structural, environmental and political factors. Given the contextual disparities in different accommodation settings, it remains unclear what exactly contributes to poorer health. It needs to be assessed how these different contextual factors are related, forming particular accommodation types with potentially different impacts on health. We hypothesize that adverse factors can accumulate, leading to health disparities in ASR across different accommodation types. This accumulation of adverse factors may explain the negative health impacts of collective accommodation.

This study had two objectives: first, to devise a broad typology of refugee accommodation by identifying clusters of characteristics which will serve as a basis for an innovative, multidimensional way of assessing the housing situation of ASR; second, to identify possible associations between the accommodation clusters and the health of ASR.

## Methods

### Dataset

We used data from the IAB-BAMF-SOEP Survey of Refugees, a cooperative longitudinal project of the Institute for Employment Research (IAB), the Migration, Integration and Asylum Research Centre at the Federal Office for Migration and Refugees (BAMF-FZ), and the Socio-Economic Panel (SOEP). A nationwide sample of initially 3,336 households and 4,527 individual respondents with refugee and asylum-seeking backgrounds were interviewed yearly form 2016. The initial questionnaire contains about 450 questions relative among others to the biography of ASR, socio-demographic data as well as post-migration stressors [9]. All respondents arrived in Germany between 2013 and 2016 [10]. Sampling method as well as an analysis on non-response can be found in Kühne et al [10]. The survey comprises self-reported variables on housing and neighbourhood characteristics as well as physical and mental health outcomes [1, 9]. This is a secondary data analysis of household panel data available to researchers in academic institutions. The data owners have obtained the consent of participants and ethical approval for the collection and use of data and no further informed consent is required [9].

We included all individuals above 18 years of age who took part in the survey in 2018 and for whom complete data was available for the variables of interest. In some cases, respondents indicated no changes in their living situation compared to the previous year (e.g., if the type of accommodation or the residential area has not changed). Where this was the case, we used data from 2017 (or from 2016 if the situation had not changed in 2017 either).

### Outcome variables

We used the mental health component score (MCS) and the physical health component score (PCS) from the SOEP version of the 12-Item Short Form (SF-12) as main outcome variables in this study. The SF-12 is a subset of the SF-36 and consists of 12 items measuring eight domains of health and health-related quality of life. MCS and PCS are two superordinate dimensions summarizing these eight domains by one single measure for mental and physical health respectively. These two main dimensions are z-transformed to a mean of 50 and a standard deviation of 10. Higher scores indicate a better mental or physical health [11, 12].

### Clustering variables

We selected the variables relative to the housing context based on an analytical framework that we have described elsewhere [13] and which briefly recall here. The framework builds on theoretical concepts of camps and social institutions. It and identifies four dimensions of ASR housing that are relevant from a health perspective: first, political factors reflecting laws and policies that prescribe, restrict or favour the housing context. Second, societal factors capturing how the dwelling is integrated into its’ physical and social environment and thus promotes or inhibits integration of the residents. Third, institutional factors that describe processes and structures inside the dwelling that may diminish residents’ empowerment and self-determination. And fourth, a dimension that adds a subjective evaluation of the overall housing context to the analysis.

We screened the IAB-BAMF-SOEP variables to identify those which covered dimensions and contextual factors developed in the analytical framework. In total, we included 20 variables for clustering and coded them as described below. Four variables related to the political dimension: asylum status (coded as: “asylum status pending”, “accepted” or “rejected”), choice of residence (whether place of residence is legally restricted to a federal state or district), the number of respondents receiving housing benefits and the number of respondents paying rent on their own. Five variables reflected the societal / environmental dimension, those were area characteristics (coded as: “residential area”, “mixed area” consisting of residential and commercial districts, or “industrial area”), perceived security in the neighbourhood (coded as: “feeling not safe” and “feeling (very) safe”), the number of respondents having no contact to locals, accessibility of public transport (bus, subway / tram (way) and train), and opportunities for leisure time activities (whether respondents do leisure time activities at least once a month). The institutional dimension was represented by seven variables: dwelling type (coded as: “temporary collective accommodation”, “permanent collective accommodation”, “multi-story buildings”, “apartment blocks” or “terraced houses / detached houses”), number of residents per household / unit, living space (in sqm), number of rooms per household / unit, furnishment (coded as “self-equipped”, “furnished” and “not specified”), number of respondents inviting friends to their dwelling at least once a month, and a variable indicating the time respondents spend in boredom per week. For the individual dimension, we included four variables: satisfaction with allocated place of residence (coded as “unsatisfied”, “neither satisfied nor unsatisfied”, “satisfied” and “N.A”, the latter category was created since this question was not applicable for respondents without residence obligations), satisfaction with the general living situation (scale from 0-10, higher scores indicate higher satisfaction), satisfaction with size of the dwelling (coded as “too small”, “suitable”, “too large” and “N.A.”, the latter again for those who were not subject to residence obligations), and sense of belonging (a scale comprised of four items measuring feelings of belonging to people and to places. Scores values ranged from 1 to 4 with higher scores indicating a higher sense of belonging).

### Sociodemographic variables

We included sociodemographic characteristics such as age, gender, country of origin (Afghanistan, Syria, Iraq, Eritrea, stateless, other countries) and a variable indicating whether respondents are either currently employed or undergoing training / education to control for differences between households in the regression analyses.

The dimensions and the included variables are shown in Table 1.

**Table 1:** Cluster characteristics of refugee accommodation in Germany, 2018

Cluster*		1	2	3	4
	n	574	360	411	190
Political dimension					
Asylum status					
*Rejected*	n (%)	18 (3.1)	44 (12.1)	1 (0.2)	10 (5.3)
*Pending*	n (%)	40 (7)	96 (26.7)	67 (16.3)	22 (11.6)
*Recognized*	n (%)	516 (89.9)	220 (61.1)	343 (83.5)	158 (83.2)
Free choice of residence	n (%)	3 (0.5)	42 (11.7)	409 (99.5)	3 (1.6)
Receiving housing allowances	n (%)	87 (15.2)	50 (13.9)	65 (15.8)	18 (9.5)
Paying rent	n (%)	82 (14.3)	67 (18.6)	149 (36.3)	15 (7.9)
**Societal / environmental dimension**					
Area type					
*Residential area*	n (%)	429 (74.7)	196 (54.4)	271 (65.9)	147 (77.4)
*Mixed area*	n (%)	141 (24.6)	97 (26.9)	138 (33.6)	39 (20.5)
*Industrial area*	n (%)	4 (0.7)	67 (18.6)	2 (0.5)	4 (2.1)
Security in the neighbourhood					
*Feeling not safe*	n (%)	22 (3.8)	35 (9.7)	23 (5.6)	1 (0.5)
No contact to locals	n (%)	284 (49.5)	253 (70.3)	175 (42.6)	80 (42.1)
Accessibility of public transport					
*Bus*	n (%)	562 (97.9)	337 (93.6)	391 (95.1)	156 (82.1)
*Subway / tram (way)*	n (%)	219 (38.2)	140 (38.9)	112 (27.3)	5 (2.6)
*Train*	n (%)	511 (89)	230 (63.9)	308 (74.9)	7 (3.7)
Leisure time activities	n (%)	166 (28.9)	75 (20.8)	159 (38.7)	34 (17.9)
**Institutional dimension**					
Dwelling type					
*Temporary collective accommodation*	n (%)		82 (22.8)		
*Permanent collective accommodation*	n (%)		271 (75.3)	2 (0.5)	
*Multi-story buildings*	n (%)	154 (26.8)	5 (1.4)	96 (23.4)	23 (12.1)
*Apartment blocks*	n (%)	317 (55.2)	2 (0.6)	227 (55.2)	102 (53.7)
*Terraced houses / detached houses*	n (%)	103 (17.9)		86 (20.9)	65 (34.2)
Number of residents per unit / household	Mean (SD)	3 (1.5)	5.1 (7.3)	3.2 (1.8)	3.2 (1.6)
Living space (in sqm)	Mean (SD)	77.6 (28.7)	35.4 (32.8)	81.7 (29)	80.9 (28.2)
Number of rooms					
*No separate rooms / units*	n (%)		95 (26.4)		
*One separate room*	n (%)	52 (9.1)	262 (72.8)	36 (8.8)	10 (5.3)
*At least two separate rooms*	n (%)	522 (90.9)	3 (0.8)	375 (91.2)	180 (94.7)
Furnishment					
*Self-equipped*	n (%)	462 (80.5)	56 (15.6)	329 (80)	137 (72.1)
*Furnished*	n (%)	89 (15.5)	301 (83.6)	72 (17.5)	41 (21.6)
*Not specified*	n (%)	23 (4)	3 (0.8)	10 (2.4)	12 (6.3)
Inviting friends	n (%)	330 (57.5)	175 (48.6)	292 (71)	126 (66.3)
Time spent in boredom (hours per week)	Mean (SD)	0.8 (1.5)	1.3 (2.5)	0.6 (1.2)	0.9 (1.8)
**Individual dimension**					
Satisfaction with living situation	Mean (SD)	8.3 (2.6)	5.4 (3.3)	8.6 (2.4)	8.3 (2.5)
Satisfaction with allocated place of residence					
*Unsatisfied*	n (%)	79 (13.8)	69 (19.2)		26 (13.7)
*Neither satisfied nor unsatisfied*	n (%)	65 (11.3)	55 (15.3)		27 (14.2)
*Satisfied*	n (%)	427 (74.4)	194 (53.9)	2 (0.5)	134 (70.5)
*Not applicable*	n (%)	3 (0.5)	42 (11.7)	409 (99.5)	3 (1.6)
Satisfaction with size of the dwelling					
*Too small*	n (%)	216 (37.6)	4 (1.1)	99 (24.1)	57 (30)
*Suitable*	n (%)	342 (59.6)	3 (0.8)	292 (71)	127 (66.8)
*Too large*	n (%)	16 (2.8)		18 (4.4)	6 (3.2)
*Not applicable*	n (%)		353 (98.1)	2 (0.5)	
Sense of belonging	Mean (SD)	3.5 (1.2)	3.3 (1.2)	3.6 (1.2)	3.5 (1)

Data based on the IAB-BAMF-SOEP study (Kühne et al., 2019). *Cluster numbering used for reference to the text and Table 2 and 3

### Statistical analysis

First, we conducted a cluster analysis with the 20 clustering variables. We used the Gower coefficient to calculate distances since this measure is also applicable if variables are scaled differently [13]. Since the number of clusters should not be predetermined but tested exploratively, we chose a hierarchal, agglomerative clustering algorithm. Using Ward’s method obtained the clearest clustering after exploring the results of other fusion algorithms. We then assessed the number of the clusters to be selected via dendrogram and scatterplot. We added the identified clusters as separate variables to the data set, representing different accommodation types. In a consequent step, we assessed bivariate associations between the clusters and health (MCS and PCS) and tested them for significance via two sample t-tests. Consequently, we performed linear mixed model regression analyses to test for multivariate associations between the accommodation clusters and physical and mental health. We controlled for individuals nested within households by adding the individual’s household ID as random effect. The sociodemographic variables served as control variables in the regression analyses. Assessing collinearity statistics as well as visually exploring the residuals showed no sign of violating the regression assumptions. We tested different models for MCS and PCS using different reference categories for the cluster-variables to explore associated impacts on the regression results (reference category was changed three times to obtain all comparisons). The two models with the clearest regression results are presented in this article (see Additional file 3 for the remaining models). P-values were adjusted for the number of hypothesis tests performed using Bonferroni correction. The initial significance level of 0.05 thus reduced to 0.017 in the multivariate analyses. All statistical analyses were run with R [14] using “cluster” [15] for performing the cluster analysis and “lme4” [16] for linear mixed model analysis.

## Results

### Sample characteristics and clustering

We included data from 1535 respondents of 1159 households in the cluster analysis, considering only cases with complete data for the clustering variables (35.3% of the total sample). Around 72% of the households interviewed in the sample consisted of only one person, 24.9% of two persons, and only a minority (≈ 3%) of three to six persons. Respondents were in mean 36 years old (SD = 10.3), 37.1% of the sample were women. Most of the sample (51.9%) originated from Syria, followed by Afghanistan and Iraq (around 14% each).

The dendrogram (Additional file 1) pointed to four clusters. The scatterplot (Additional file 2) also showed a slight bend at the point of four clusters but also would have supported the selection of two or three clusters. We therefore selected four clusters (named for convenience 1 to 4 below and in tables) to identify small-scaled differences between clusters and to keep heterogeneity within the clusters as low as possible. Cluster 1 comprised of 574 cases, Cluster 2 of 360 cases, Cluster 3 of 411 cases and Cluster 4 comprised 190 cases.

### Cluster characteristics

Cluster characteristics are displayed in detail in Table 1. Overall, respondents living in private accommodation spread over three different clusters (1, 3 and 4) whereas those from collective accommodation types formed a separate cluster (Cluster 2).

*Political dimension*. In all clusters, respondents predominantly had accepted asylum claims with percentages of more than 80% in clusters 1, 3 and 4, and 61% in Cluster 2. Consequently, rates of asylum seekers and those with rejected asylum claims were the highest in Cluster 2. While respondents in clusters 1, 2 and 4 were predominantly subject to residence obligations, almost all Cluster 3 residents (99.5%) could themselves decide where to live. In this cluster, also more than one third were able to pay the rent on their own. In the other clusters, this proportion was at most half as high.

*Societal-environmental dimension*. Respondents across all clusters most often lived in residential areas, with percentages ranging from 54 % in Cluster 2 to 77% in Cluster 4. Cluster 2 showed the highest proportion of respondents living in industrial areas (20%). Additionally, slightly more people of Cluster 2 did not feel safe in their neighbourhoods compared to the other clusters. Overall, though, security issues were of only little concern. More distinct differences could be found regarding the contact to locals. While in Cluster 2 more than 70% stated to have no contact to locals in the neighbourhood, minimum half of the respondents in the other clusters meet local neighbours at least sporadically. In terms of leisure time activities, Cluster 3 residents were more active than residents from the other clusters. Almost 40% of them stated to do leisure time activities at least once a month (compared to 20% to 29% in the other clusters). In terms of public transport, not surprisingly busses formed the mode of transport which was best accessible in all clusters, followed by trains and subways / tram (way). In Cluster 4, access to all modes of transport was remarkably lower than in the other clusters. Particularly striking was the poor accessibility of trains (3.7%) and subways / tram (way) (2.6%) in this cluster.

*Institutional dimension.* Most frequent dwelling type in the clusters 1, 3 and 4 were apartment blocks in which more than half of the respondents lived. Compared to clusters 1 and 3, Cluster 4 residents more frequently lived in detached and terraced houses (34%) and less frequently in multi-story buildings (12%). In contrast, almost all of the residents from Cluster 2 either lived in temporary (23%) or permanent collective accommodation centres (75%). Accordingly, Cluster 2 showed major differences in terms of living space. With on average 35.4 (SD = 32.8) sqm and around five people (SD = 7.3) per unit, Cluster 2 residents had less space available than residents from the private accommodation clusters, especially from the clusters 3 and 4 in which around three residents (SD ≈ 1.8) shared more than 80 sqm (SD ≈ 29) on average. While in all three private accommodation clusters more than 90% of the residents had two or more rooms at their disposal, one in four Cluster 2 residents did not even have a separate living unit. They also differed from the other clusters in terms of how they occupy their time. They least frequently invited friends to their dwelling (less than half of the residents stated to invite friends at least once a month) and spent more time in boredom (only in Cluster 2 boredom time exceeded one hour per week).

*Individual dimension*. Overall, we found the lowest satisfaction and belonging scores in Cluster 2. While in the private accommodation clusters the general satisfaction with the living situation was in mean above 8 / 10 (SD ≤ 2.6), mean score in Cluster 2 was 5.4 (SD = 3.3). Among all who were subject to residence obligations (cluster 1, 2 and 4), dissatisfaction with the allocated place of residents was slightly higher in Cluster 2 with around 20% compared to around 14% in Cluster 1 and 4. The same applies to the respondents’ sense of belonging which was slightly lower in Cluster 2. Further, Cluster 1 residents most frequently (almost 40%) perceived their dwelling as too small, while residents from cluster 3 and 4 were more often satisfied with the size of their dwelling (for people from collective accommodation, i.e., Cluster 2, this information was not available).

### Bivariate health associations

The clusters identified showed some differences in the health status of their residents as Table 2 illustrates. Generally, all clusters showed slightly higher values for PCS than for MCS. Mental health was poorest in Cluster 2 with a MCS of 47.1 (SD = 11.9), this score was significantly lower than in the other clusters at p < 0.05 (t_(df = 890)_ = -2.6; p =0.009). In the clusters 3 and 4, the mean MCS was highest and even slightly exceeded a value of 50. In terms of physical health, we found the lowest PCS in Cluster 4 with a mean of 50.7 (SD = 10.3). Clusters 2 and 3 showed the highest scores with values reaching almost 54. The difference between Cluster 4 and the clusters 2 and 3 were statistically significant (t_(df = 518)_ = -3.4; p < 0.001).

**Table 2:** Bivariate associations between mental health and physical health of refugees, Germany, 2018

Cluster*				
	1	2	3	4
**MCS**				
Mean (SD)	49.2 (11.4)	47.1* (11.9)	50.5 (10.3)	50.1 (10.8)
Valid n (%)	553 (96.4)	339 (98.7)	406 (98.8)	181 (95.3)
**PCS**				
Mean (SD)	51.9 (10.2)	53.9 (10.3)	53.8 (9.3)	50.7** (10.3)
Valid n (%)	553 (96.4)	339 (98.7)	406 (98.8)	181 (95.3)

Note: *MCS* Mental health component score, *PCS* Physical health component score

*MCS significant compared to cluster 1,2 and 3 (at p < 0.05); ** PCS significant compared to cluster 2 and 3 (at p < 0.05)

Data based on the IAB-BAMF-SOEP study (Kühne et al., 2019). *Cluster numbering used for reference to the text and Table 1 and 3

### Multivariate health associations

An overview of the results of the regression analyses is provided in Table 3. In Model 1, MCS was regressed against cluster and the control variables age, gender, origin and current work / education. In Model 2, PCS was regressed against cluster with the same control variables. The 1475 individuals included in the analyses were nested within 1114 households. For Model 1, household units resembled stronger than for Model 2 with an intraclass correlation of 0.27 compared to 0.03 respectively. For Model 1, we found that belonging to one of the private accommodation clusters (1, 3 and 4) was significantly associated with better mental health compared to belonging to Cluster 2, adjusting for age, gender, origin and current work / education. Among the private accommodation clusters, the regression coefficients of the clusters 3 and 4 were the highest (b = 3.63 and b = 3.7 respectively, p < 0.001). Further, female gender was significantly associated with poorer mental health while originating from Eritrea and being employed or under training showed a significant, positive association with mental health.

**Table 3:** Results of linear mixed model regression analysis of health of refugees, Germany, 2018

		Model 1: MCS		Model 2: PCS	
		b (SE)	p-value	b (SE)	p-value
**Fixed parts:**					
Constant		47.82** (1.35)	0.0000	67.89** (1.11)	0.0000
Age		-0.04 (0.03)	0.1561	-0.36** (0.02)	0.0000
Gender (female)		-1.98** (0.59)	0.0009	-3.65** (0.51)	0.0000
Origin	Syria	*Ref*		*Ref*	
	Afghanistan	-0.33 (0.94)	0.7241	0.37 (0.72)	0.6130
	Eritrea	3.61** (1.27)	0.0045	2.53** (1.01)	0.0125
	Iraq	1.08 (0.94)	0.2512	-1.02 (0.72)	0.1586
	Stateless	0.44 (1.93)	0.8182	-0.31 (1.54)	0.8413
	Other countries	1.17 (1.03)	0.2546	0.50 (0.81)	0.5386
Work / Education		1.68** (0.68)	0.0140	0.83 (0.56)	0.1409
Cluster*	Cluster 1	2.78** (0.83)	0.0008	-1.37* (0.60)	0.0227
	Cluster 2	*Ref*		-1.14 (0.71)	0.1064
	Cluster 3	3.63** (0.90)	0.0001	*Ref*	
	Cluster 4	3.70** (1.10)	0.0008	-2.49** (0.82)	0.0024
**Random parts:**					
N (households)		1114		1114	
ICC		0.27		0.03	
Observations		1475		1475	
R^2^		0.29		0.24	

Notes: MCS, mental health component score; PCS, physical health component score; ICC = intraclass correlation; Ref, reference category; * significant at p < 0.05, ** significant at p <0.017 (Bonferroni correction). Data based on IAB-BAMF-SOEP study (Kühne et al., 2019).*Cluster numbering used for reference to the text and Table 2 and 3

In Model 2, Cluster 4 showed a significant, negative association with physical health compared to Cluster 2 (b = -2.49, p = 0.002). The other two clusters failed to yield significant results (for Cluster 1 this applied only after adjusting the level of significance due to multiple testing). As in Model 1, female gender and originating from Eritrea were significant control variables.

In additional regression models (Additional file 3) we found significant negative associations with mental health for cluster 2 when changing the reference category to cluster 1,3 or 4. For physical health, we found significant positive associations for cluster 3 when using cluster 1 and 4 as reference, but not when using cluster 2 as reference.

## Discussion

We conducted a cluster analysis to first analyse the housing situation of ASR in Germany, and then to assess accommodation-associated health disparities using population-based data of the IAB-BAMF-SOEP study. We found four different accommodation clusters, three of them reflecting different types of private accommodation, while one cluster comprised all respondents living in collective accommodation. The latter (Cluster 2) differed from the other clusters in terms of space, area, level of restrictions, level of social connections and the level of respondent satisfaction: residents had less space at their disposal, more often lived in industrial areas (but generally with good accessibility to public transport), were less often in contact to locals, rarely conducted dedicated leisure time activities, and showed the poorest satisfaction with their living situation. The higher rates of respondents whose asylum claim is pending or rejected and the high rate of respondents with residence obligations may indicate a rather restrictive living situation.

Cluster 1 showed characteristics of an urban setting with high scores for respondent satisfaction, although residence restrictions were in place. Public transport access was the best of all clusters. The high rate of access to subways and tram (way) and the fact that almost one in three people lived in (often too small) flats of multi-story buildings points to more urbanized areas.

Cluster 3 and 4 both comprised accommodation types that provided sufficient space. In both clusters people felt quite satisfied with their living situation and reported good social connections with locals. But in contrast to Cluster 3, Cluster 4 accommodation apparently was more frequently located in rural areas. On the one hand, this may enable respondents to live in more spacious building types (such as terraced houses and detached houses) but on the other hand may also deprive them of good connectivity: they had the poorest access to public transport and also least often reported leisure time activities, which could point to generally fewer opportunities for leisure time activities. In Cluster 3, in contrast, public transport was better accessible and rates of people performing leisure time activities were higher. The major difference between Cluster 3 and the other clusters, though, was that almost all respondents could themselves decide where to live and they also paid their rent on their own more frequently. This could indicate a higher level of self-dependency in this cluster.

In terms of health disparities, belonging to the collective accommodation cluster was significantly associated with poorer mental health than belonging to any of the private accommodation clusters. Given that distribution to facilities is quasi-random, accommodation thus plays a role in the production of mental health inequalities. When unfavourable living conditions accumulate - like in the collective accommodation cluster - these conditions can act simultaneously with more deleterious effects for health [17]. This is supported by previous research pointing to the negative health impact of collective accommodation [3, 4].

Asylum status must be taken into account here, as well, since people with pending or rejected asylum status more frequently live in these settings (as Cluster 2 demonstrated). The asylum process is considered as a post-migration stressor itself [1, 4] and may thus attribute to the negative health impacts in collective accommodation. The asylum status is closely related to housing, though, since it restricts or enables to make own housing decisions. Holding a residence permit seems to improve mental health outcomes, presumably due to the optimized living conditions that come along with it [17]. Related to that, aspects of the housing environment that pertain to the exercise of control are considered as significant predictors of mental health [18, 19]. In Cluster 3, residents seem to have the largest level of control of their housing situation (own choice of residence, recognized refugee status, paying rent on their own) while Cluster 4 residents seem to have the lowest level of control.

Physical health was significantly lower in Cluster 4 compared to clusters 2 and 3 in bivariate analysis and cluster 3 in multivariate analysis. The main difference to the other clusters was the poor accessibility of public transport and related to that, the overall impression of a rurally located accommodation type. This could lead to the assumption that access to health services is more difficult for residents and, as a result, health problems are less likely to be treated and may eventually worsen. In support of this assumption, Biddle et al. [20] also pointed to potential equity issues in terms of health care access for ASR living in rural areas with an increased level of unmet needs of medical services. Hahn et al. [21] identified similar equity issues in providing access to health care for ASR. Since both studies were conducted in collective accommodation centres, the results do not provide a clear explanation for the poorer physical health outcomes in Cluster 4 (as a private accommodation cluster) but could at least partially support our assumption.

### Strength and limitations

Previous research has put little emphasis on a comparison of the housing situation of ASR living in private and collective accommodation settings in this detail. We included not only mere physical factors of housing but also related social, political and individual factors that determine the housing situation of ASR. In previous studies among ASR, postmigration stressors (including housing conditions) have often been assessed as stand-alone factors. In this study, we have analysed the combined role of these multidimensional factors. We demonstrated that certain housing conditions cluster, leading to different accommodation types that are differently associated with health.

It must be noted that the cross-sectional design of the study does not allow conclusions on causality of the associations identified. For example, it remains unclear whether the conditions in Cluster 2 caused mental problems or whether poor mental health rather fostered negative experiences and perceptions. As Fergusson et al. [22] pointed out, life satisfaction is reciprocally associated with mental health. Similar needs to be taken into account here. Longitudinal approaches would be required investigating whether health problems are a consequence of housing.

Due to missing values in the data of certain cluster variables, we were only able to analyse 35.3% of the total sample. Although we imputed data from previous years where possible, the variables area type and dwelling type contained high numbers of missing data (both around 44.2%). In subsequent analysis we tested how results changed when removing the two variables from the cluster analysis. Since the results revealed a similar clustering and the two variables were considered as relevant for assessing the housing situation, we left them in the analysis, being aware that this can affect the accuracy of our estimations.

## Conclusions

This study underlines that collective accommodation settings clearly differ from private accommodation settings in terms of structures, the physical environment and the social opportunities attached to it, legal restrictions resulting from the asylum process, and the level of individual’s housing satisfaction. Our findings support that collective accommodation negatively affects mental health of ASR. We have shown that unfavourable conditions cluster and are associated with negative mental health but not with physical health. Most favourable is a setting in apartments blocks, located in a residential area that was chosen by the resident, provides good accessibility to public transport and enables good social connections. Health differences also exist in the private accommodation setting. However, more research would be necessary regarding the association between housing and physical health of ASR since we were not able to provide clear explanations for the differences in physical health found in the clusters. But still, the clusters identified in this study can be part of a new approach to analysing housing of ASR that goes beyond the mere distinction between private and collective accommodation and helps to identify health associations in a more differentiated way.

## Supplementary Information

**Additional file 1.** Dendrogram cluster analysis.

**Additional file 2.** Scatterplot cluster analysis.

**Additional file 3.** Regression results with changed reference categories.

## Data Availability

The data that support the findings of this study are available from the German Institute of Economic Research (DIW). All information about the public availability of the data can be found here: https://www.diw.de/de/diw_01.c.615551.de/forschungsbasierte_infrastruktureinrichtung__sozio-oekonomisches_panel__soep.html

## Abbreviations

ASR: Asylum seekers and refugees
BAMF-FZ: Migration, Integration and Asylum Research Centre at the Federal Office for Migration and Refugees
IAB: Institute for Employment Research
MCS: Mental health component score
PCS: Physical health component score
SF-12: Short-Form 12
SOEP: Socio-Economic Panel
