# Supplementary figures and images for: Association between housing and health of refugees and asylum seekers in Germany: explorative cluster and mixed model analysis
Source: BMC Public Health. 2022 Jan 8;22:48. doi: 10.1186/s12889-021-12458-1 (PMC8742454; doi:10.1186/s12889-021-12458-1)

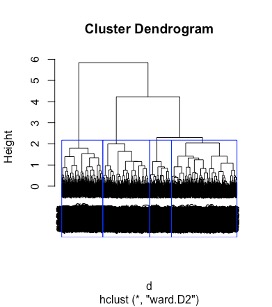

Supplement: Supplementary file 1 — Additional file 1. Dendrogram cluster analysis. [file 12889_2021_12458_MOESM1_ESM.jpg]

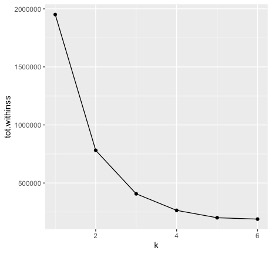

Supplement: Supplementary file 2 — Additional file 2. Scatterplot cluster analysis. [file 12889_2021_12458_MOESM2_ESM.jpg]
