# Supplementary material for: Association between housing and health of refugees and asylum seekers in Germany: explorative cluster and mixed model analysis
Source: BMC Public Health. 2022 Jan 8;22:48. doi: 10.1186/s12889-021-12458-1 (PMC8742454; doi:10.1186/s12889-021-12458-1)
Supplement: Supplementary file 3 — Additional file 3. Regression results with changed reference categories. [file 12889_2021_12458_MOESM3_ESM.docx]

**Additional file 3: Regression results with changed reference categories**

| **MCS** | | | | | | | |
| --- | --- | --- | --- | --- | --- | --- | --- |
|  |  | **Ref = 1** |  | **Ref = 3** |  | **Ref = 4** |  |
|  |  | **b (SE)** | **p-value** | **b (SE)** | **p-value** | **b (SE)** | **p-value** |
| Constant |  | 50.62** (1.32) | 0.000 | 51.45** (1.36) | 0.000 | 51.51** (1.48) | 0.000 |
| Age |  | -0.04 (0.03) | 0.156 | -0.04 (0.03) | 0.156 | -0.04 (0.03) | 0.156 |
| Gender (female) | | -1.98** (0.59) | 0.001 | -1.98** (0.59) | 0.001 | -1.98** (0.59) | 0.001 |
| Origin | Syria | *Ref* |  | *Ref* |  | *Ref* |  |
|  | Afghanistan | -0.33 (0.94) | 0.724 | -0.33 (0.94) | 0.724 | -0.33 (0.94) | 0.724 |
|  | Eritrea | 3.61** (1.27) | 0.005 | 3.61** (1.27) | 0.005 | 3.61** (1.27) | 0.005 |
|  | Iraq | 1.08 (0.94) | 0.251 | 1.08 (0.94) | 0.251 | 1.08 (0.94) | 0.251 |
|  | Stateless | 0.44 (1.93) | 0.818 | 0.44 (1.93) | 0.818 | 0.44 (1.93) | 0.818 |
|  | other | 1.17 (1.03) | 0.255 | 1.17 (1.03) | 0.255 | 1.17 (1.03) | 0.255 |
| Work / Education | | 1.68** (0.68) | 0.014 | 1.68** (0.68) | 0.014 | 1.68** (0.68) | 0.014 |
| Cluster | Cluster 1 | *Ref* |  | - 0.83 (0.77) | 0.281 | - 0.90 (0.99) | 0.366 |
|  | Cluster 2 | - 2.80** (0.83) | 0.000 | - 3.63** (0.90) | 0.000 | - 3.63**(1.10) | 0.001 |
|  | Cluster 3 | 0.83 (0.77) | 0.280 | *Ref* |  | - 0.06 (1.06) | 0.951 |
|  | Cluster 4 | 0.9 (0.99) | 0.370 | 0.07 (1.05) | 0.951 | *Ref* |  |
| **PCS** | | | | | | | |
|  |  | **Ref = 1** |  | **Ref = 2** |  | **Ref = 4** |  |
|  |  | **b (SE)** | **p-value** | **b (SE)** | **p-value** | **b (SE)** | **p-value** |
| Constant |  | 66.52** (1.08) | 0.000 | 66.74** (1.11) | 0.000 | 65.40** (1.20) | 0.000 |
| Age |  | -0.36** (0.02) | 0.000 | -0.36** (0.02) | 0.000 | -0.36** (0.02) | 0.000 |
| Gender (female) | | -3.65** (0.51) | 0.000 | -3.65** (0.51) | 0.000 | -3.65** (0.51) | 0.000 |
| Origin | Syria | *Ref* |  | *Ref* |  | *Ref* |  |
|  | Afghanistan | 0.37 (0.72) | 0.613 | 0.37 (0.72) | 0.613 | 0.37 (0.72) | 0.613 |
|  | Eritrea | 2.53** (1.01) | 0.013 | 2.53** (1.01) | 0.013 | 2.53** (1.01) | 0.013 |
|  | Iraq | -1.02 (0.72) | 0.159 | -1.02 (0.72) | 0.159 | -1.02 (0.72) | 0.159 |
|  | Stateless | -0.31 (1.54) | 0.841 | -0.31 (1.54) | 0.841 | -0.31 (1.54) | 0.841 |
|  | other | 0.50 (0.81) | 0.539 | 0.50 (0.81) | 0.539 | 0.50 (0.81) | 0.539 |
| Work / Education | | 0.83 (0.56) | 0.141 | 0.83 (0.56) | 0.141 | 0.83 (0.56) | 0.141 |
| Cluster | Cluster 1 | *Ref* |  | - 0.22 (0.65) | 0.735 | 1.12 (0.77) | 0.148 |
|  | Cluster 2 | 0.22 (0.65) | 0.735 | *Ref* |  | 1.34 (0.85) | 0.116 |
|  | Cluster 3 | 1.37* (0.60) | 0.023 | 1.14 (0.71) | 0.106 | 2.49** (0.81) | 0.002 |
|  | Cluster 4 | - 1.12 (0.77) | 0.148 | - 1.34 (0.85) | 0.116 | *Ref* |  |
| *Notes: MCS, mental health component score; PCS, physical health component score;* Ref, reference category; ** significant at p < 0.05, ** significant at p <0.017 (Bonferroni correction).*  Data based on IAB-BAMF-SOEP study (Kühne et al., 2019). | | | | | | | |
